# Masculinity, Lifestyle Factors, and Andropause‐Related Symptoms in Pakistani Men: A Cross‐Sectional Study

**DOI:** 10.1002/hsr2.73305

**Published:** 2026-09-25

**Authors:** Sehar Adnan, Sidra Afzal

**Affiliations:** ^1^ School of Psychology, Faculty of Health Sciences and Wellbeing University of Sunderland Sunderland UK

**Keywords:** Ageing males' symptoms, andropause‐related symptoms, lifestyle health, masculinity contingency, men's health, Pakistani men

## Abstract

**Background and Aims:**

Andropause‐related symptoms may reflect biological ageing, lifestyle behaviours, and culturally shaped understandings of masculinity. Evidence examining lifestyle health, masculinity contingency, and self‐reported Ageing Males' Symptoms (AMS) among Pakistani men is limited. This study examined their associations and whether masculinity contingency moderated the lifestyle–symptom relationship.

**Methods:**

A cross‐sectional correlational study included 150 Pakistani men aged 30–56 years who completed an online Qualtrics survey. Data were collected in 2024. Measures included the Lifestyle and Habits Questionnaire‐Brief, AMS scale, and Masculinity Contingency Scale (MCS). Analyses comprised descriptive statistics, reliability analysis, Pearson correlations, multivariable linear regression, and moderation analysis using IBM SPSS Statistics version 29.0.1.0.

**Results:**

Lifestyle health was inversely associated with AMS scores (r = −0.365, *p* < 0.001). In multivariable regression, lifestyle health remained negatively associated with AMS scores (B = −1.180, 95% CI [−1.658, −0.701], *p* < 0.001), and the model explained 14.3% of the variance. MCS Boost showed a positive bivariate association with AMS scores (r = 0.178, *p* = 0.03), whereas MCS Threat was not significantly associated with AMS scores. In the moderation model, MCS Boost was positively associated with AMS scores (B = 0.401, 95% CI [.095, 0.707], *p* = 0.01), but neither MCS Boost nor MCS Threat moderated the lifestyle–AMS association. Age was not significantly associated with AMS scores.

**Conclusion:**

Among Pakistani men aged 30–56 years, healthier lifestyle scores were associated with lower self‐reported ageing‐related symptom burden. Masculinity contingency did not moderate the lifestyle–symptom association, although MCS Boost showed a positive association with symptom scores. These cross‐sectional findings identify lifestyle health as a relevant consideration in culturally sensitive men's health promotion. Longitudinal studies incorporating broader age ranges, clinical indicators, and culturally grounded qualitative work are needed to clarify temporal, biological, and cultural influences on symptom burden.

## 1 Introduction

Masculinity is a socially and culturally shaped construct that influences how men understand health, ageing, vulnerability, and help‐seeking. Masculine norms that emphasise strength, autonomy, endurance, sexual performance, economic responsibility, and emotional restraint can affect whether men recognise symptoms as health concerns, communicate distress, or seek timely support [1]. For symptoms involving fatigue, low mood, sexual functioning, or reduced vitality, these expectations may be particularly relevant because symptoms may be interpreted as weakness or decline rather than manageable health concerns.

In Pakistan, masculinity is often embedded within family roles, social responsibility, expectations of provision and authority, and concerns about respectability [2, 3, 4]. Men may be expected to act as protectors, decision‐makers, and financial providers while maintaining emotional control. These expectations may encourage resilience and responsibility, but may also discourage open discussion of physical or psychological difficulties. Health problems affecting sexual performance, energy, mood, or confidence may therefore carry social and personal sensitivity.

Although these symptoms are sometimes discussed using the term andropause, this terminology remains debated because clinical late‐onset hypogonadism requires both symptoms and biochemical assessment, and age‐related testosterone decline is variable across men [5, 6, 7]. In this article, the term andropause‐related symptoms is used cautiously to describe self‐reported physical, psychological, and sexual symptoms measured with the Ageing Males' Symptoms (AMS) scale [8]. Participants were not clinically diagnosed with andropause or late‐onset hypogonadism, and testosterone levels were not measured.

The psychological and social significance of ageing male symptoms is not limited to biological change. Symptoms may be experienced through social expectations, cultural beliefs, family responsibilities, and personal identity [1, 2]. A man who interprets fatigue, reduced sexual desire, or low mood as a shameful sign of ageing may respond differently from a man who understands these experiences as health concerns that can be discussed with professionals.

Lifestyle health was conceptualised as a multidimensional pattern of behaviours and health‐related practices that may be associated with physical, psychological, and sexual wellbeing across adulthood. In this study, it was assessed using the Lifestyle and Habits Questionnaire‐Brief (LHQ‐B), comprising health and exercise, psychological health, substance use, nutrition, environmental concern, social concern, accident prevention, and sense of purpose [9].

Several domains have plausible links with AMS‐measured symptom burden. Physical inactivity and smoking have been associated with greater AMS symptom severity [10], while depressive and anxiety symptoms overlap substantially with AMS scores [11]. Nutrition and metabolic health may also be relevant: higher AMS scores have been associated with insulin resistance, higher body mass index, and problematic eating behaviours [12, 13].

Other LHQ‐B domains, including social concern, sense of purpose, environmental concern, and accident prevention, reflect broader aspects of health orientation, although their individual relationships with AMS scores remain less directly studied. Across adulthood, lifestyle practices may accumulate or interact with stress, health status, and age‐related physiological changes. Accordingly, the present study examines overall lifestyle health as a multidimensional correlate of self‐reported AMS symptom burden rather than assuming that each domain contributes equally or through a single causal pathway.

Masculinity contingency theory provides a useful framework for examining how men's self‐worth may depend on perceived conformity to masculine expectations. The Masculinity Contingency Scale (MCS) distinguishes between threat and boost dimensions: threat reflects reduced self‐worth when masculine standards are perceived as unmet, while boost reflects increased self‐worth when masculinity is affirmed [14]. These dimensions may influence how men interpret ageing‐related symptoms involving fatigue, sexual functioning, emotional distress, or reduced strength.

Previous research on ageing male symptoms and androgen deficiency has focused largely on Western or clinical populations [6, 15, 16], while studies from South Asian and Pakistani contexts remain limited. Existing Pakistani research has more often considered masculinity, fertility, family roles, or broader health concerns separately rather than examining the combined relationship among lifestyle health, masculinity contingency, and ageing male symptoms [2, 3, 4]. This gap is important because the meaning of masculinity is culturally situated.

The present study therefore examined lifestyle health, masculinity contingency, and self‐reported Ageing Males' Symptoms among Pakistani men. By studying these variables together, the research aimed to contribute to men's health literature in a non‐Western context and to identify whether lifestyle health is associated with symptom burden. The study also examined whether masculinity contingency threat and boost were directly associated with symptom scores and whether they moderated the relationship between lifestyle health and ageing male symptoms.

An integrated approach is useful because ageing‐related symptoms may coexist with psychological and social factors, while masculine norms can shape health interpretation and help‐seeking [1, 17].

### 1.1 Study Aims and Hypotheses

This study examined the relationships among lifestyle health, masculinity contingency, and self‐reported Ageing Males' Symptoms among Pakistani men. Specifically, the study tested: (1) whether lifestyle health was associated with Ageing Males' Symptoms scores; (2) whether masculinity contingency threat and masculinity contingency boost were directly associated with Ageing Males' Symptoms scores; and (3) whether masculinity contingency threat and boost moderated the association between lifestyle health and Ageing Males' Symptoms scores.

**H1:** *Higher lifestyle health scores would be associated with lower Ageing Males' Symptoms scores.*

**H2:** *Masculinity contingency threat and masculinity contingency boost would be associated with Ageing Males' Symptoms scores.*

**H3:** *Masculinity contingency threat and masculinity contingency boost would moderate the association between lifestyle health and Ageing Males' Symptoms scores*.

## 2 Methods

### 2.1 Design

This study used a cross‐sectional correlational design to examine associations among lifestyle health, masculinity contingency, and self‐reported ageing male symptoms among Pakistani men.

The study is reported in accordance with the Strengthening the Reporting of Observational Studies in Epidemiology (STROBE) Statement for cross‐sectional studies [18].

### 2.2 Participants and Recruitment

Participants were recruited through convenience sampling. Recruitment information and the survey link were circulated through Facebook and WhatsApp to reach Pakistani men aged 30–70 years living in different cities of Pakistan. These platforms were used because they allowed access to a geographically dispersed sample and enabled participants to complete the survey privately. Although the eligibility range was 30–70 years, the final analytic sample consisted of 150 complete responses from men aged 30–56 years. Most participants were from Lahore, Karachi, and Multan, with additional responses from other areas of Pakistan. Apart from age and geographic location, the study did not collect additional demographic or clinical characteristics suitable for inclusion in the regression models.

### 2.3 Eligibility Criteria

Participants were eligible if they were Pakistani men aged 30–70 years, able to read and understand English, had internet access, and voluntarily agreed to complete the online survey. The 30–70‐year eligibility range was selected a priori to capture variation in self‐reported symptoms from the 30's into later adulthood. AMS symptoms are nonspecific and do not define a discrete age of onset or independently diagnose androgen deficiency. AMS‐based assessment has been used in men aged 30–39 years, whereas age‐related testosterone decline and clinically defined late‐onset hypogonadism become increasingly relevant with advancing age [6, 15, 19]. The selected range was therefore intended to examine andropause‐related symptom burden across adulthood rather than to identify clinically diagnosed late‐onset hypogonadism. Individuals were excluded if they were female, younger than 30 years, or reported clinically diagnosed conditions affecting hormone production or clinically diagnosed mental health disorders. These criteria were used to focus the study on adult Pakistani men who could complete the English‐language measures and provide informed consent online.

### 2.4 Sample Size

The target sample size was estimated using G*Power, based on an approximate correlation of 0.20 between Ageing Males' Symptoms and masculinity contingency, power of 0.80, and alpha of 0.05 [20], yielding a target of approximately 200 participants. A total of 200 responses were received. Following data screening and removal of incomplete responses, 150 complete responses were retained for analysis. The smaller analytic sample may have reduced statistical power, particularly for detecting interaction effects.

### 2.5 Measures

Lifestyle health was measured using the Lifestyle and Habits Questionnaire‐Brief (LHQ‐B [9]), which assesses eight domains: health and exercise, psychological health, substance use, nutrition, environmental concern, social concern, accident prevention, and sense of purpose. Each domain score was classified using the published male‐specific Bottom, Middle, and Top interpretive ranges and coded 1, 2, or 3, respectively. The eight coded domain scores were summed to create a study‐derived lifestyle‐health composite ranging from 8 to 24, with higher scores indicating healthier lifestyle patterns. The lifestyle scale demonstrated acceptable internal consistency in the present sample (α = 0.811).

Self‐reported ageing‐related symptoms were measured using the 17‐item Ageing Males' Symptoms scale (AMS [8]). The AMS assesses physical, psychological, and sexual symptoms commonly discussed in relation to male ageing. Higher scores indicate greater symptom severity. In this study, the AMS was used to assess symptom burden and was not used to diagnose andropause, late‐onset hypogonadism, or testosterone deficiency. The scale showed excellent internal consistency in the present sample.

Masculinity contingency was measured using the 10‐item Masculinity Contingency Scale (MCS [14]). The scale comprises two five‐item dimensions: MCS Threat, reflecting reductions in self‐worth when masculine expectations are perceived as unmet, and MCS Boost, reflecting increases in self‐worth when masculinity is affirmed. Items are rated from 1 (strongly disagree) to 7 (strongly agree). Items within each dimension were summed to produce separate MCS Threat and MCS Boost scores ranging from 5 to 35, with higher scores indicating greater threat‐ or boost‐related masculinity contingency, respectively. The subscale scores were analysed as continuous variables. The overall 10‐item MCS demonstrated excellent internal consistency in the present sample (α = 0.910).

### 2.6 Procedure and Ethics

Ethical approval was obtained from the University of Sunderland Research Ethics Committee (Application No. 026552). Data were collected in 2024. Participants viewed study information online before beginning the survey. The information explained the study purpose, voluntary nature of participation, confidentiality procedures, and the right to withdraw. Electronic informed consent was obtained before participants completed the measures. Participants created unique alphanumeric codes to support withdrawal while protecting identity. Responses were collected through Qualtrics and later exported to Excel for initial organisation. Data were then cleaned and analysed using SPSS version 29.0.1.0. Throughout the study, participant anonymity and confidentiality were maintained.

### 2.7 Data Analysis

Data were analysed using IBM SPSS Statistics version 29.0.1.0. Descriptive statistics were used to summarise participant characteristics and scale scores. Internal consistency was assessed using Cronbach's alpha. Associations among lifestyle health, masculinity contingency, and Ageing Males' Symptoms scores were examined using Pearson correlations. Pearson correlation coefficients were reported with 95% confidence intervals calculated using Fisher's z‐transformation. A multivariable linear regression model examined the associations of lifestyle health and Age with Ageing Males' Symptoms scores. Moderation analysis used mean‐centred lifestyle health, MCS Threat, and MCS Boost scores, following standard guidance for testing interaction effects in multiple regression [21]. The model included age, the main effects of lifestyle health, MCS Threat, and MCS Boost, and two product terms representing lifestyle health × MCS Threat and lifestyle health × MCS Boost. Predictors were entered simultaneously on the basis of the specified analytical models; no data‐driven variable‐selection procedure was used. The a priori significance level was α = 0.05, and all inferential tests were two‐sided. Regression diagnostics included assessment of linearity and homoscedasticity using residual plots, normality of residuals using Q–Q plots, multicollinearity using variance inflation factors, and influential observations using Cook's distance, leverage, studentized residuals, and DFBETAs. Analyses were conducted using complete cases; incomplete responses were excluded during data screening, leaving no missing values among variables included in the regression models. The correlation, regression, and moderation analyses were hypothesis‐driven. The sensitivity analysis for the potentially influential observation was conducted as a supplementary robustness check. No exploratory subgroup analyses were conducted.

For descriptive reporting of symptom severity, AMS total scores were categorised as no/little symptoms (17–26), mild (27–36), moderate (37–49), or severe (≥ 50), according to established AMS severity ranges [8].

## 3 Results

The final sample comprised 150 Pakistani men aged 30–56 years (M = 43.34, SD = 7.89). Although eligibility extended to 70 years, no participant in the analytic sample was older than 56 years. Participants were most commonly from Lahore, Karachi, and Multan, with the remaining participants from other cities and regions of Pakistan. Participant characteristics, scale descriptive statistics, and internal consistency estimates are presented in Table 1.

**Table 1:** Participant Characteristics, Scale Descriptive Statistics, and Internal Consistency Estimates.

Variable/scale	Value
Total analytic sample	150
Age, years	M = 43.34, SD = 7.89; median = 43.50, IQR = 37.00–50.00
Age range, years	30–56
Lahore	59/150 (39.3%)
Karachi	18/150 (12.0%)
Multan	16/150 (10.7%)
Other cities/regions	57/150 (38.0%)
Ageing Males' Symptoms score	M = 36.02, SD = 11.57; median = 35.00, IQR = 26.25–45.00
Lifestyle health score	M = 17.13, SD = 3.66; median = 17.00, IQR = 15.00–20.00
MCS Threat score	M = 21.23, SD = 7.53; median = 22.00, IQR = 16.00–27.00
MCS Boost score	M = 22.87, SD = 7.07; median = 23.00, IQR = 20.00–28.00
AMS scale	α = 0.912
Lifestyle scale	α = 0.811
MCS overall (10 items)	α = 0.910

*Note:* α = Cronbach's alpha; AMS = Ageing Males' Symptoms; IQR = interquartile range; M = mean; MCS = Masculinity Contingency Scale; SD = standard deviation. MCS Threat and MCS Boost are summed scores with a possible range of 5–35.

Based on the AMS severity classification, 38/150 (25.3%) participants reported no/little symptoms, 45/150 (30.0%) mild symptoms, 43/150 (28.7%) moderate symptoms, and 24/150 (16.0%) severe symptoms.

Pearson correlations were conducted to examine associations among the main study variables. As shown in Table 2, lifestyle health was significantly associated with lower Ageing Males' Symptoms scores. MCS Boost showed small positive associations with both Ageing Males' Symptoms and lifestyle health, whereas MCS Threat was not significantly associated with either variable.

**Table 2:** Pearson Correlations Among Main Study Variables.

Variable pair	r	95% CI	*p*
Lifestyle health and Ageing Males' Symptoms	−0.365	[−0.496, −0.218]	< 0.001
MCS Threat and Ageing Males' Symptoms	0.115	[−0.047,0.270]	0.16
MCS Boost and Ageing Males' Symptoms	0.178	[0.018,0.329]	0.03
MCS Threat and lifestyle health	0.082	[−0.079, 0.239]	0.32
MCS Boost and lifestyle health	0.177	[0.017,0.328]	0.03

*Note:* r = Pearson correlation coefficient; CI = confidence interval; MCS = Masculinity Contingency Scale.

The bivariate correlation findings supported H1. Higher lifestyle‐health scores were associated with lower Ageing Males' Symptoms scores (r = −0.365, 95% CI [−0.496, −0.218], *p* < 0.001). Higher MCS Boost scores showed a small positive association with Ageing Males' Symptoms scores (r = 0.178, 95% CI [.018, 0.329], *p* = 0.03), whereas MCS Threat scores were not significantly associated with symptom scores (r = 0.115, 95% CI [−0.047, 0.270], *p* = 0.16); accordingly, H2 received partial support. Higher MCS Boost scores were also positively associated with lifestyle health (r = 0.177, 95% CI [.017, 0.328], *p* = 0.03), whereas MCS Threat scores were not significantly associated with lifestyle health (r = 0.082, 95% CI [ − 0.079, 0.239], *p* = 0.32).

Diagnostic checks did not indicate substantial violations of linearity, residual normality, homoscedasticity, or multicollinearity. One high‐leverage observation was identified in the moderation model; sensitivity analysis excluding this case did not alter the substantive pattern or statistical significance of the main and interaction effects, and the observation was therefore retained. A multivariable linear regression examined the associations of lifestyle health and Age with Ageing Males' Symptoms scores. Lifestyle health was significantly associated with lower Ageing Males' Symptoms scores, B = −1.180, SE = 0.242, β = −0.373, 95% CI [ − 1.658, −0.701], *p* < 0.001. Age was not significantly associated with Ageing Males' Symptoms scores, B = −0.142, SE = 0.112, β = −0.097, 95% CI [ − 0.364, 0.080], *p* = 0.21. The model explained 14.3% of the variance in Ageing Males' Symptoms scores, R^2^ = 0.143, adjusted R^2^ = 0.131. Full model estimates are presented in Table 3.

**Table 3:** Multivariable Linear Regression Model for Ageing Males' Symptoms Scores.

Predictor	B	SE	β	95% CI	*p*
Intercept	62.372	6.710	—	[49.112, 75.632]	< 0.001
Lifestyle health	−1.180	0.242	−0.373	[−1.658, −0.701]	< 0.001
Age	−0.142	0.112	−0.097	[−0.364, 0.080]	0.21

*Note:* Outcome variable = Ageing Males' Symptoms score. B = unstandardized regression coefficient; β = standardised regression coefficient; CI = confidence interval; SE = standard error. The model was significant, F (2, 147) = 12.24, *p* < 0.001, R^2^ =.143, adjusted R^2^ = 0.131. *N* = 150.

**Figure 1:**
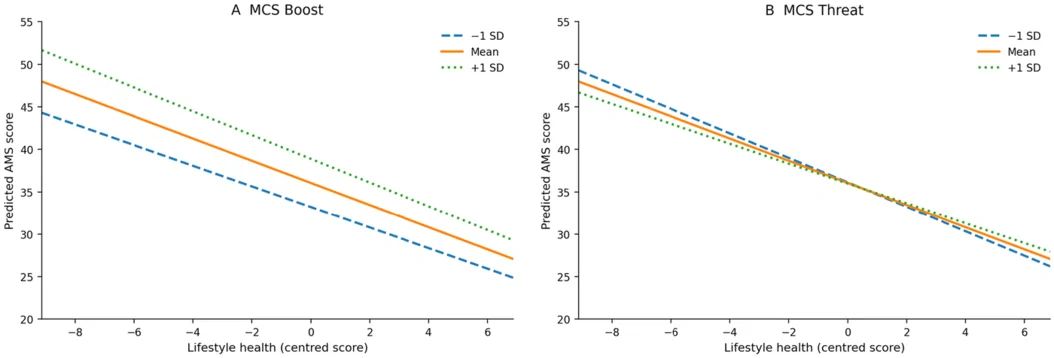
Predicted Ageing Males' Symptoms (AMS) scores across mean‐centred lifestyle health at −1 SD, mean, and +1 SD of continuous MCS Boost (Panel A) and MCS Threat (Panel B). Predictions were estimated with age held at the sample mean (43.34 years). MCS = Masculinity Contingency Scale; SD = standard deviation. The near‐parallel slopes are consistent with the nonsignificant lifestyle health × MCS Boost and lifestyle health × MCS Threat interaction terms.

Moderation analysis examined whether MCS Threat and MCS Boost moderated the association between lifestyle health and Ageing Males' Symptoms scores. As shown in Table 4, higher lifestyle‐health scores remained significantly associated with lower Ageing Males' Symptoms scores. Higher MCS Boost scores were positively associated with symptom scores (B = 0.401, 95% CI [.095, .707], *p* = 0.01), whereas MCS Threat was not significantly associated (B = −.009, 95% CI [ − .298, 0.280], *p* = 0.95). Neither the lifestyle health × MCS Boost interaction (*p* = 0.73) nor the lifestyle health × MCS Threat interaction (*p* = 0.64) was statistically significant, providing no evidence that either MCS dimension moderated the lifestyle–symptom association. Accordingly, H3 was not supported. Figure 1 illustrates the predicted patterns.

**Table 4:** Moderation Analysis of Ageing Males' Symptoms Scores.

Predictor	B	SE	β	95% CI	*p*
Intercept	40.901	4.858	—	[31.298, 50.504]	< 0.001
Age	−0.112	0.110	−0.077	[−0.330, 0.106]	0.31
Lifestyle health, centred	−1.307	0.245	−0.414	[−1.791, −0.823]	< 0.001
MCS Boost, centred	0.401	0.155	0.245	[0.095,0.707]	0.01
MCS Threat, centred	−0.009	0.146	−0.006	[−0.298,0.280]	0.95
Lifestyle health × MCS Boost	−0.013	0.038	−0.033	[−0.088,0.062]	0.73
Lifestyle health × MCS Threat	0.018	0.039	.046	[−0.058,0.095]	0.64

*Note:* Outcome = Ageing Males' Symptoms score. B = unstandardized regression coefficient; β = standardised regression coefficient; CI = confidence interval; MCS = Masculinity Contingency Scale; SE = standard error. F (6, 143) = 6.01, *p* < 0.001, R^2^ = 0.201, adjusted R^2^ =.168. *N* = 150.

## 4 Discussion

The findings suggest that lifestyle health is a meaningful correlate of self‐reported andropause‐related symptom burden among Pakistani men. Symptoms such as fatigue, low mood, sleep disturbance, reduced vitality, and sexual concerns are nonspecific and cannot be attributed to hormonal ageing on the basis of self‐reported AMS scores alone [6, 7]. In this sample, poorer lifestyle health was associated with higher symptom scores, supporting a broader biopsychosocial interpretation in which health behaviours, stress, cultural expectations, and self‐reported ageing‐related symptoms may overlap. Age was not significantly associated with AMS scores in the multivariable analyses. However, the restricted and relatively young age distribution limits interpretation of age‐related patterns. Elevated AMS scores in this sample may also reflect psychological distress, sleep disturbance, lifestyle factors, chronic health conditions, or other nonspecific symptoms rather than age‐related endocrine change.

The inverse association between lifestyle health and Ageing Males' Symptoms is consistent with population‐based evidence that modifiable health behaviours may accompany AMS symptom burden. Corrêa et al. [10] found that smoking and physical inactivity were associated with greater symptom burden among Brazilian men aged 40 years and older. AMS scores, however, are not specific to lifestyle behaviours or hormonal ageing. Higher scores have also been associated with insulin resistance [12] and psychological distress [11], while Hamanoue et al. [12] found no significant association between total AMS scores and testosterone levels. These findings support a broader biopsychosocial interpretation rather than a specific causal pathway.

In the Pakistani context, lifestyle advice may be more acceptable when it recognises family responsibility, privacy, respectability, and reluctance to discuss sensitive symptoms [2, 3, 4]. Health education that links lifestyle behaviours with everyday functioning, vitality, and family participation may therefore be more acceptable than messages framed around weakness or decline.

Masculinity contingency showed a more complex pattern. MCS Threat was not significantly associated with Ageing Males' Symptoms scores, which differs from previous research linking masculinity contingency threat with adverse psychological outcomes. Burkley et al. [14] reported that the threat dimension was generally more strongly associated with negative personal and social outcomes than the boost dimension, while McDermott et al. [22] found that masculinity contingency threat was positively associated with depressive and social anxiety symptoms among U.S. university men. The absence of a comparable association in the present sample may reflect differences in outcome, age, cultural context, or the way masculinity‐related self‐worth is expressed among Pakistani men.

Higher MCS Boost scores showed a small positive bivariate association with Ageing Males' Symptoms scores and remained positively associated in the multivariable moderation model. Although a positive association was observed in the continuous‐score analyses, its magnitude was small and the cross‐sectional design precludes causal interpretation. Previous research suggests that MCS Threat and MCS Boost may operate differently, with threat more consistently associated with adverse outcomes and boost sometimes linked with positive self‐evaluative functioning [14]. The present positive association between MCS Boost and symptom burden therefore requires replication. Greater investment in masculine affirmation might heighten awareness of symptoms involving vitality, physical functioning, or sexual wellbeing, but the current data cannot establish this mechanism.

Neither MCS Threat nor MCS Boost moderated the association between lifestyle health and Ageing Males' Symptoms scores. Directly comparable evidence is limited because previous masculinity‐contingency research has primarily examined psychological and social outcomes rather than whether masculinity contingency modifies associations between lifestyle health and ageing‐male symptoms [14, 22]. The nonsignificant interactions should therefore be interpreted as an absence of evidence for moderation in this sample rather than evidence that masculinity contingency cannot influence symptom experiences. Cultural differences in the meaning and expression of masculinity, together with the restricted age range and sample size, may also have limited the detection of interaction effects.

These results should not be interpreted as evidence that masculinity is unimportant. Rather, masculinity may operate through culturally specific pathways that were not fully captured by the quantitative measure used in this study, such as family responsibility, emotional restraint, sexual health stigma, social respectability, and reluctance to seek help [1, 2].

The findings also have implications for clinical communication. Healthcare professionals working with Pakistani men may need to normalise conversations about fatigue, sleep, mood, sexual health, stress, and ageing‐related changes. Men may be more willing to seek advice when symptoms are framed as common health experiences rather than as threats to masculinity [1].

Lifestyle health emerged as a significant correlate, but it explained only part of the variation in symptoms. Ageing male symptoms may also be shaped by hormonal status, chronic illness, medication use, relationship factors, psychological distress, and access to healthcare.

### 4.1 Limitations and Future Directions

Several limitations should be noted. Convenience sampling, online recruitment, and English‐language measures may limit generalisability, particularly to men without internet access, lower English literacy, or rural and socioeconomically diverse backgrounds. All measures were self‐reported, creating potential for recall and social desirability bias, especially for masculinity and sexual symptoms.

The cross‐sectional design precludes causal inference, and the analytic sample was aged 30–56 despite eligibility extending to 70; findings may therefore not represent older Pakistani men. Marital status, education, economic status, body mass index, chronic disease status, and medication use were not collected and therefore could not be included in the regression models; residual confounding from these factors cannot be excluded. Although the AMS, LHQ‐B, and MCS demonstrated acceptable internal consistency in this sample, internal consistency does not establish cultural validity. The study did not independently assess whether these English‐language measures adequately capture culturally specific meanings of masculinity, ageing, lifestyle, or sexual health among Pakistani men.

Future research should include larger, more age‐diverse Pakistani samples, particularly men in older age groups not represented in the present sample, and longitudinal designs to examine temporal relationships between lifestyle health and symptom burden. Studies should incorporate testosterone levels, body mass index, chronic disease status, medication use, and mental health history. Future work should also prioritise Urdu‐language or bilingual cultural adaptation and psychometric validation of the AMS, LHQ‐B, and MCS in Pakistani men, examining whether their structure and meaning are appropriate within the local cultural context rather than relying on internal consistency alone. If culturally adapted MCS versions still fail to capture locally salient dimensions of masculinity, development of a culturally grounded measure should be considered.

Qualitative research should examine how Pakistani men interpret ageing‐related physical, psychological, and sexual symptoms; how masculinity is understood in relation to provider responsibility, family authority and obligation, respectability, emotional restraint, sexual wellbeing, and help‐seeking; and how these meanings shape symptom disclosure and healthcare engagement. Particular attention should be given to family and intergenerational expectations, financial responsibility, perceptions of masculine threat or affirmation, and the stigma attached to sexual or emotional difficulties. Exploring variation across age groups, urban and rural settings, socioeconomic circumstances, and family contexts would help determine which aspects of masculine identity are culturally specific and which may be captured adequately by existing measures. Mixed‐methods research could then integrate these culturally grounded accounts with quantitative symptom and lifestyle data.

### 4.2 Implications

In clinical practice, healthcare professionals working with Pakistani men could incorporate brief, non‐stigmatising assessment of physical activity, diet, sleep, smoking or other substance use, psychological distress, and sexual wellbeing when men present with fatigue, reduced vitality, sleep disturbance, low mood, or sexual concerns. Where appropriate, management may include support for regular physical activity, smoking cessation, healthier dietary practices, sleep hygiene, and stress management, together with referral for medical assessment when symptoms are persistent or severe. These recommendations should be viewed as areas for assessment and health promotion rather than as treatments demonstrated by the present cross‐sectional study. Evidence linking physical inactivity and smoking with greater AMS symptom burden supports the relevance of these modifiable behaviours [10].

Community‐based health promotion may benefit from culturally sensitive messaging that frames lifestyle health around energy, functioning, wellbeing, and participation in family life rather than weakness or loss of masculinity. Private opportunities to discuss sexual symptoms, emotional distress, and ageing‐related concerns may be important where disclosure is difficult. Community organisations, primary‐care services, and culturally appropriate digital materials may provide suitable channels for health information. Such approaches should be sensitive to culturally shaped expectations surrounding family roles, emotional restraint, masculinity, and help‐seeking among Pakistani men [2, 3, 4].

## 5 Conclusion

Among Pakistani men aged 30–56 years in this cross‐sectional study, healthier lifestyle scores were associated with lower self‐reported Ageing Males' Symptoms scores. MCS Boost showed a small positive bivariate association with symptom scores and remained positively associated with symptom scores in the multivariable moderation model, but neither MCS Boost nor MCS Threat moderated the lifestyle‐symptom relationship. These findings support a cautious biopsychosocial interpretation of andropause‐related symptom burden and highlight lifestyle health as a relevant consideration in men's health promotion, alongside culturally sensitive communication and further research using clinically assessed and age‐diverse Pakistani samples.

## Author Contributions

**Sehar Adnan:** conceptualization, investigation, writing – original draft, methodology, validation, visualization, writing – review and editing, formal analysis, project administration, software. **Sidra Afzal:** writing – review and editing, supervision.

## Funding

The authors have nothing to report.

## Ethics Statement

Ethical approval for this study was obtained from the University of Sunderland Research Ethics Committee, United Kingdom (Application No. 026552). Data were collected in 2024. All participants received information about the study before participation and provided electronic informed consent. Participation was voluntary, and participant confidentiality and anonymity were maintained throughout the study.

## Conflicts of Interest

The authors declare no conflicts of interest.

## Transparency Statement

Sehar Adnan affirms that this manuscript is an honest, accurate, and transparent account of the study being reported; that no important aspects of the study have been omitted; and that any discrepancies from the study as planned have been explained.

## Data Availability

The data that support the findings of this study are available from the corresponding author upon reasonable request. The data are not publicly available because they contain information collected from human participants and are subject to ethical and confidentiality restrictions. No separate analytic code or syntax file is available because the analyses were conducted interactively using IBM SPSS Statistics. The research instruments used in the study are identified and cited in the Methods section and should be obtained from their original sources, subject to any applicable permissions or licensing requirements.
